# Reticuloendotheliosis virus and avian leukosis virus subgroup J synergistically increase the accumulation of exosomal miRNAs

**DOI:** 10.1186/s12977-018-0427-0

**Published:** 2018-07-03

**Authors:** Defang Zhou, Jingwen Xue, Shuhai He, Xusheng Du, Jing Zhou, Chengui Li, Libo Huang, Venugopal Nair, Yongxiu Yao, Ziqiang Cheng

**Affiliations:** 1 College of Veterinary Medicine, Shandong Agricultural University, Tai’an, 271018 China; 2 The Pirbright Institute & UK-China Centre of Excellence on Avian Disease Research, Pirbright, Ash Road, Guildford, Surrey, GU24 0NF UK

**Keywords:** Reticuloendotheliosis virus, Avian leukosis virus subgroup J, Exosomal miRNAs, Synergistic infection

## Abstract

**Background:**

Co-infection with avian leukosis virus subgroup J and reticuloendotheliosis virus induces synergistic pathogenic effects and increases mortality. However, the role of exosomal miRNAs in the molecular mechanism of the synergistic infection of the two viruses remains unknown.

**Results:**

In this study, exosomal RNAs from CEF cells infected with ALV-J, REV or both at the optimal synergistic infection time were analysed by Illumina RNA deep sequencing. A total of 54 (23 upregulated and 31 downregulated) and 16 (7 upregulated and 9 downregulated) miRNAs were identified by comparing co-infection with two viruses, single-infected ALV-J and REV, respectively. Moreover, five key miRNAs, including miR-184-3p, miR-146a-3p, miR-146a-5p, miR-3538 and miR-155, were validated in both exosomes and CEF cells by qRT-PCR. GO annotation and KEGG pathway analysis of the miRNA target genes showed that the five differentially expressed miRNAs participated in virus-vector interaction, oxidative phosphorylation, energy metabolism and cell growth.

**Conclusions:**

We demonstrated that REV and ALV-J synergistically increased the accumulation of exosomal miRNAs, which sheds light on the synergistic molecular mechanism of ALV-J and REV.

**Electronic supplementary material:**

The online version of this article (10.1186/s12977-018-0427-0) contains supplementary material, which is available to authorized users.

## Background

Viral synergism occurs commonly in nature when co-infection of two or more unrelated viruses invades the same host. Both reticuloendotheliosis virus (REV) and avian leukosis virus subgroup J (ALV-J), as two oncogenic retroviruses, consist of a set of retroviral genes, env, pol, gag and LTR, and mainly induce reticuloendotheliosis and myelocytomas, respectively [1, 2]. Due to similar transmission routes, co-infection with ALV-J and REV can readily occur [3, 4] and spreads very rapidly [5–7]. Co-infection of ALV-J and REV induces more serious pathogenic effects, such as immunosuppression, growth retardation, accelerated neoplasia progression, secondary infection in chickens [3, 5], and increased mortality. Exosomes, intraluminal vesicles ranging approximately 30-100 nm in diameter secreted by live cells, have emerged as important molecules for intercellular communication that are involved in both normal and pathophysiological conditions, such as lactation, immune response and neuronal function, and in the development and progression of diseases, such as liver disease, neurodegenerative diseases and cancer [8–17]. Exosomes contain a wide variety of proteins, lipids, RNAs, non-transcribed RNAs, microRNAs and small RNAs to induce a diverse range of functions from intercellular communication to tumour proliferation [14, 18]. As useful biomarkers, exosomes are also helpful for exploring the synergistic mechanisms of co-infection with ALV-J and REV.

MicroRNAs (miRNAs) constitute a large family of small noncoding RNAs functioning as major regulators of gene expression in cancer development [19–21]. The mature miRNA regulates spatio-temporal gene expression by binding to a seed region in the 3′ untranslated region (UTR) but may also bind to the 5′ UTR of target mRNA to enhance mRNA translation inhibition or degradation, resulting in decreased protein expression [22, 23]. Although miRNAs occupy negligible genomic space, their influence on a myriad of physiological processes, such as growth, differentiation, apoptosis, host–pathogen interactions and energy metabolism, is indubitable and relevant in tumour progression [24–30]. A growing number of studies have certified that miRNAs, including miR-122, miR-29b, miR-34a and miR-155, are key regulators of tumourigenesis, especially in viral synergistic infection [31–33]. However, the role of miRNA-mediated regulation of co-infection with ALV-J and REV remains unknown.

In the present study, to reveal the roles of miRNA profiles in the synergistic infection with ALV-J and REV, exosomal miRNAs were extracted from CEF infected with ALV-J, REV or both at the optimal synergistic infection time to analyse by Illumina RNA deep sequencing. The key miRNAs obtained from deep sequencing were validated in exosomes and CEFs by qRT-PCR. Furthermore, the affected miRNA–mRNA interactions and associated biological processes were defined by integrated target prediction analyses.

## Results

### Synergistic infection of ALV-J and REV increases virus replication in CEF

**Fig. 1:**
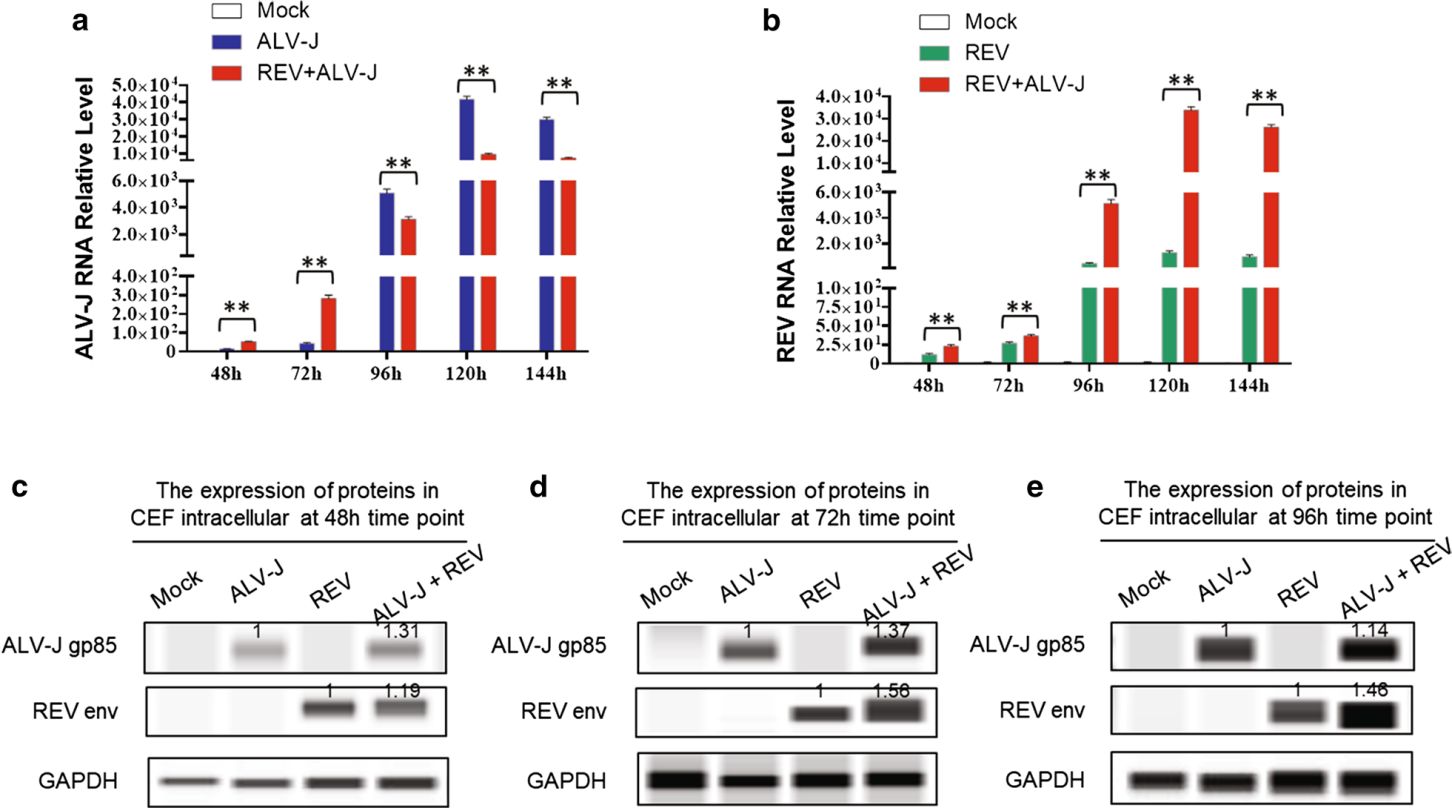
Co-infection of ALV-J and REV promoted viral replication in CEFs. **a** REV increased the ALV-J RNA level at 48 hpi and 72 hpi while the ALV-J RNA levels in the co-infection group were decreased compared to those in the singly infected ALV-J at 96 hpi, 122 hpi and 144 hpi. The data represent the mean ± SEM determined from three independent experiments (n = 3), with each experiment containing three technical replicates. Compared with the single-infection group: **P < 0.01. **b** ALV-J increased the REV RNA level at 48, 72, 96, 122 and 144 hpi. The data represent the mean ± SEM determined from three independent experiments (n = 3), with each experiment containing three technical replicates. Compared with the single-infection group: **P < 0.01. **c** ALV-J synergized with REV to promote viral protein levels in CEF cells at 48 hpi detected by western blot with an anti-ALV-J gp85 antibody and anti-REV env antibody. **d** ALV-J synergized with REV to promote viral protein levels in CEF cells at 72 hpi detected by western blot with an anti-ALV-J gp85 antibody and anti-REV env antibody. **e** ALV-J synergized with REV to promote viral protein levels in CEF cells at 96 hpi detected by western blot with an anti-ALV-J gp85 antibody and anti-REV env antibody

To determine the best synergistic co-infection time of ALV-J and REV in vitro, we built an in vitro model of CEFs co-infected with ALV-J and REV and conducted viral RNA transcription analysis. The qRT-PCR results showed that both ALV-J and REV RNA levels in the co-infection group were increased significantly compared to those in the single infection groups at 48 hpi and 72 hpi and reached the highest peak at 72 hpi (Fig. 1a and 1b). However, the ALV-J RNA levels in the co-infection group were dramatically declined compared to those in the single infection groups at 96 hpi, 122 hpi and 144 hpi (Fig. 1a) while ALV-J still synergized with REV to promote viral replication at 96 hpi, 120 hpi and 144 hpi (Fig. 1b). The viral protein expression levels were detected by western blot with anti-gp85 of ALV-J or anti-env of REV, and the results showed that the synergistic infection of ALV-J and REV increased each virus protein expression at 48 hpi, 72 hpi and 96 hpi (Fig. 1c, 1d and 1e). Consequently, co-infection of ALV-J and REV leads to the enhancement of viral transcription and protein expression in vitro.

### Deep sequence analysis of exosomal miRNAs

**Fig. 2:**
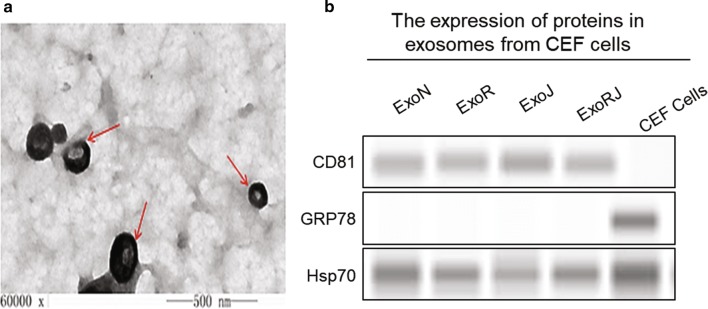
Extraction and identification of exosomes from CEFs. **a** The morphological characterization of exosomes under electron microscopy. The arrow indicates exosomes, 40–150 nm in size. **b** The purities of exosomes were also verified by western blot with anti-Hsp70, anti-Grp78 or anti-CD81

To explore the miRNA profile of co-infection with ALV-J and REV, the exosomal miRNA from CEFs infected with ALV-J, REV or both were analysed using miRNA whole-genome sequencing at 72 hpi. We obtained the exosomes successfully and did not have any impurities by transmission electron microscopy. The photo showed that the exosomes had the typical goblet structure, and the size varied between 40 and 150 nm (Fig. 2a). The purities of exosomes were also verified by western blot with anti-Hsp70, anti-Grp78 and anti-CD81. Western blot analysis showed that both exosomal protein samples were positive for CD81, a known exosomal protein, and negative for Grp78, an endoplasmic reticulum marker (Fig. 2b).

**Fig. 3:**
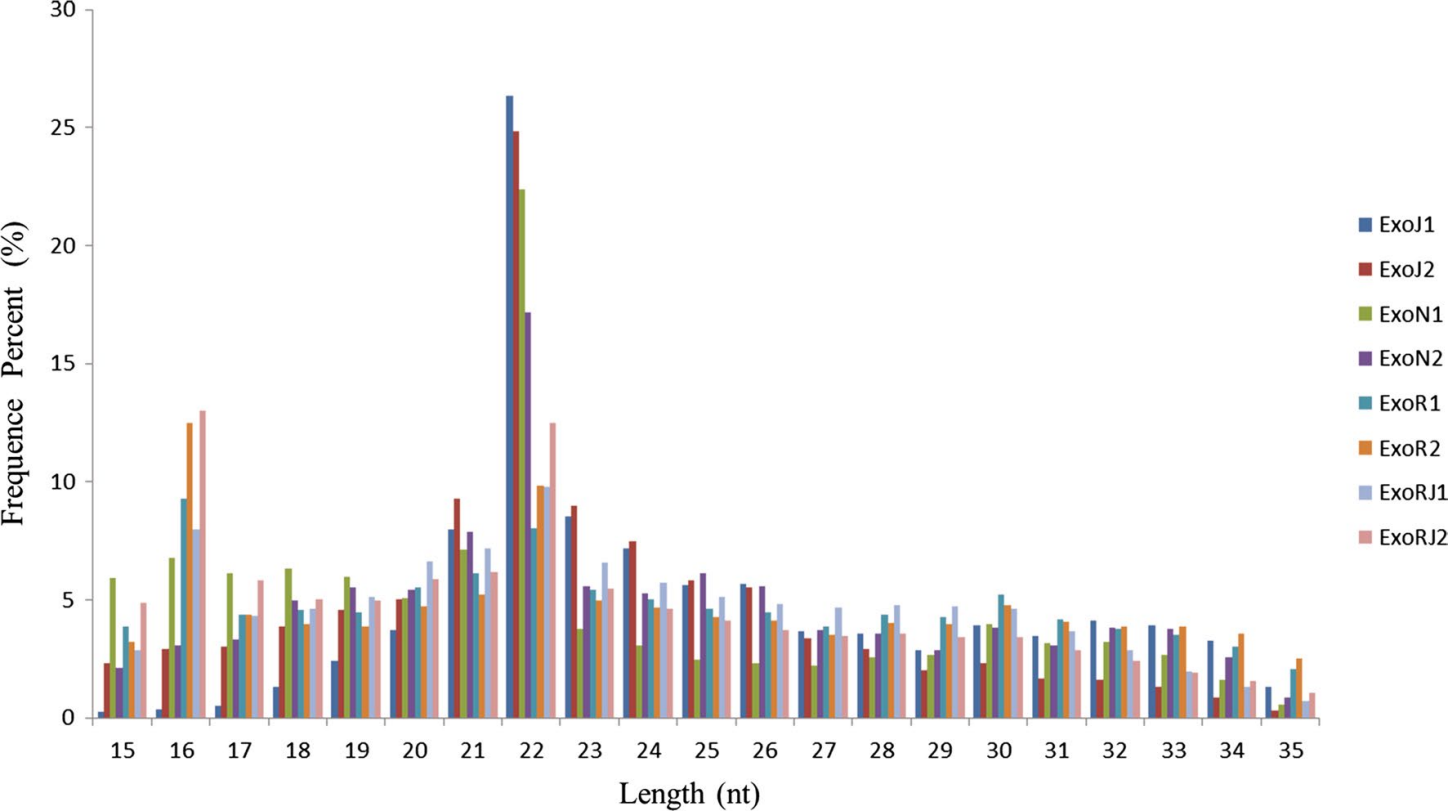
Length distributions of the clean reads of the sequences. The abundances of the sequences in the peaks are shown. The peaks in CEF-uninfected, infected with ALV-J, REV, or both were at 21–23 nt

Further, 4 miRNA libraries were generated from exosomes of normal CEFs (ExoN), CEFs infected with ALV-J (ExoJ), REV (ExoR), and both (ExoRJ), and each group had two duplicates. In total, approximately 18,482,128 to 27,087,654 high-quality raw reads were obtained from the exosome libraries (Table 1). The selected reads from these libraries mapped well to the chicken genome, and the perfect match rates were 42.42% and 78.75% of the total reads (Table 1). After filtering the empty adaptors, low-quality sequences and single-read sequences, almost 100% clean reads of 15-35 nt were selected for further analysis. The remainder of the sequences were found to be other types of RNA, including rRNA, snRNA, snoRNA, tRNA, and noncoding RNA. The size distribution of small RNAs is summarized in Fig. 3. The results showed that the peaks in CEF-uninfected, infected ALV-J, REV, or both were concentrated at 21–23 nt.

**Table 1:** Summary of deep sequencing data for small RNAs in ExoN, ExoJ, ExoR, or ExoRJ

Categories	ExoJ1	ExoJ2	ExoN1	ExoN2	ExoR1	ExoR2	ExoRJ1	ExoRJ2
Raw reads	32,802,706	33,930,695	34,958,139	32,703,162	40,626,293	39,142,674	35,806,903	47,791,565
Clean reads	25,528,138	22,734,959	18,482,128	25,269,659	26,451,518	19,409,373	27,087,654	26,375,518
Map to genome percent (%)	72.43	77.02	67.15	78.75	61.58	58.72	44.3	42.42
Exon-antisense	993,966	1,018,809	418,741	761,889	516,146	323,086	414,847	346,494
Intron-sense	82,595	67,299	67,235	94,664	78,494	59,812	75,956	60,438
Intron-antisense	66,891	66,383	48,160	84,863	56,699	33,887	63,692	54,035
miRNAs	5,564,323	5,391,541	3,120,429	3,314,531	1,154,633	583,363	1,048,558	714,469

### Identification of ALV-J and REV synergistic activated exosomal miRNAs in CEFs

**Fig. 4:**
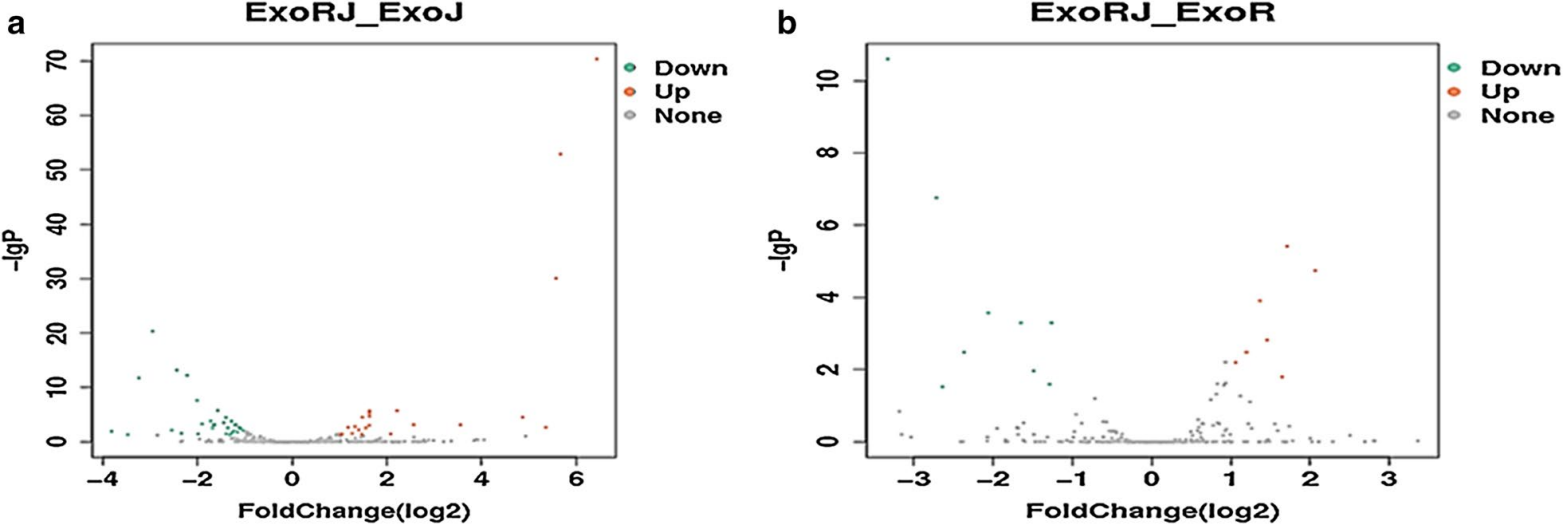
The volcano plots of miRNAs by comparing co-infection with two viruses with ALV-J-infected (**a**) and REV-infected (**b**) groups. The x and y axes represent the fold change of the relative expression (log2) and the relative expression of the miRNAs, respectively

To compare the differentially expressed miRNAs between co-infected and the two single virus infected CEFs, the differentially expressed miRNAs were mapped to the miRNA precursors of the reference species in the Sanger miRBase 21.0 database [34]. Using a P-value < 0.05 and │log2 (fold change)│≥ 1 as the cut-off values, a total of 54 (23 upregulated and 31 downregulated) and 16 (7 upregulated and 9 downregulated) miRNA genes were identified by comparing ExoRJ with ExoJ and ExoR, respectively (Fig. 4, Tables 2, 3).

**Table 2:** Differentially expressed microRNAs between ExoRJ and ExoJ

miRNA name	FC	*P* value	FDR	Up/down
gga-miR-92-3p	2.77	4.45×10^−3^	0.04	Up
gga-miR-456-3p	2.8	9.21×10^−7^	3.18×10^−5^	Up
gga-miR-429-3p	86	8.65×10^−74^	4.47×10^−71^	Up
gga-miR-375	29.1	8.16×10^−7^	3.02×10^−5^	Up
gga-miR-3538	3.1	4.48×10^−7^	1.78×10^−5^	Up
gga-miR-3529	3.08	5.52×10^−5^	1.06×10^−3^	Up
gga-miR-30c-2-3p	2.05	4.18×10^−3^	0.04	Up
gga-miR-30a-3p	3.1	1.04×10^−7^	4.47×10^−6^	Up
gga-miR-223	2.06	3.87×10^−3^	0.039	Up
gga-miR-222b-3p	11.8	3.48×10^−5^	7.50×10^−4^	Up
gga-miR-221-3p	4.65	3.67×10^−8^	1.89×10^−6^	Up
gga-miR-2131-3p	2.66	4.54×10^−4^	6.35×10^−3^	Up
gga-miR-184-3p	5.94	2.85×10^−5^	7.01×10^−4^	Up
gga-miR-1684a-3p	2.51	8.90×10^−5^	1.64×10^−3^	Up
gga-miR-155	50.8	5.03×10^−56^	1.30×10^−53^	Up
gga-miR-146a-5p	41	1.37×10^−4^	2.37×10^−3^	Up
gga-miR-146a-3p	47.7	5.35×10^−33^	9.22×10^−31^	Up
gga-miR-144-3p	2.95	1.79×10^−4^	2.72×10^−3^	Up
gga-miR-142-5p	2.27	1.33×10^−4^	2.37×10^−3^	Up
gga-miR-1416-5p	4.24	3.94×10^−3^	0.039	Up
gga-miR-1329-5p	2.41	2.72×10^−3^	0.03	Up
gga-let-7c-5p	2.06	3.45×10^−3^	0.036	Up
gga-miR-6548-5p	0.071	9.08×10^−4^	0.011	Down
gga-miR-460a-3p	0.382	3.29×10^−3^	0.035	Down
gga-miR-455-5p	0.336	3.03×10^−8^	1.74×10^−6^	Down
gga-miR-455-3p	0.38	1.13×10^−6^	3.64×10^−5^	Down
gga-miR-34a-3p	0.322	3.22×10^−5^	7.24×10^−4^	Down
gga-miR-32-3p	0.172	5.48×10^−4^	7.45×10^−3^	Down
gga-miR-30e-5p	0.305	4.99×10^−6^	1.51×10^−4^	Down
gga-miR-30d	0.45	1.44×10^−3^	0.017	Down
gga-miR-30b-5p	0.215	6.92×10^−15^	5.96×10^−13^	Down
gga-miR-301b-3p	0.434	3.98×10^−5^	8.02×10^−4^	Down
gga-miR-301a-3p	0.487	6.54×10^−4^	8.68×10^−3^	Down
gga-miR-2954	0.185	6.53×10^−16^	6.75×10^−14^	Down
gga-miR-26a-3p	0.199	2.51×10^−3^	0.028	Down
gga-miR-219b	0.472	2.5×10^−4^	3.63×10^−3^	Down
gga-miR-2131-5p	0.412	5.73×10^−6^	1.65×10^−4^	Down
gga-miR-20a-3p	0.253	3.68×10^−3^	0.038	Down
gga-miR-203a	0.09	5.58×10^−3^	0.049	Down
gga-miR-199-5p	0.313	1.88×10^−4^	2.78×10^−3^	Down
gga-miR-193b-3p	0.13	3.44×10^−23^	4.45×10^−21^	Down
gga-miR-190a-5p	0.268	2.00×10^−5^	5.18×10^−4^	Down
gga-miR-18a-5p	0.465	1.66×10^−4^	2.69×10^−3^	Down
gga-miR-181a-3p	0.439	3.01×10^−5^	7.08×10^−4^	Down
gga-miR-1729-5p	0.106	2.66E−14	1.99×10^−12^	Down
gga-miR-16-5p	0.414	2.19×10^−3^	0.025	Down
gga-miR-148a-5p	0.462	1.64×10^−4^	2.69×10^−3^	Down
gga-miR-146c-5p	0.317	4.03×10^−5^	8.02×10^−4^	Down
gga-miR-146c-3p	0.404	5.18×10^−3^	0.0461	Down
gga-miR-146b-5p	0.368	1.09×10^−5^	2.97×10^−4^	Down
gga-miR-1451-5p	0.249	3.64×10^−10^	2.35×10^−8^	Down
gga-miR-10b-3p	0.426	8.66×10^−4^	0.011	Down
gga-miR-101-2-5p	0.39	1.76×10^−4^	2.72×10^−3^	Down

*FC* fold change, *FDR* false discovery rate (corrected *P* value)

**Table 3:** Differentially expressed microRNAs between ExoRJ and ExoR

miRNA name	FC	p value	FDR	Up/down
gga-miR-146a-3p	4.18	1.66×10^−7^	1.84×10^−5^	Up
gga-miR-155	3.27	2.62×10^−8^	3.87×10^−6^	Up
gga-miR-184-3p	3.13	5.41×10^−4^	1.59×10^−2^	Up
gga-let-7a-2-3p	2.74	3.08×10^−5^	1.51×10^−3^	Up
gga-miR-458a-3p	2.29	8.31×10^−5^	3.34×10^−3^	Up
gga-miR-429-3p	2.08	1.89×10^−4^	6.44×10^−3^	Up
gga-miR-3538	0.42	9.19×10^−6^	5.08×10^−4^	Down
gga-miR-1454	0.41	1.04×10^−3^	2.56×10^−2^	Down
gga-miR-460b-5p	0.36	3.45×10^−4^	1.09×10^−2^	Down
gga-miR-133c-3p	0.32	8.86×10^−6^	5.08×10^−4^	Down
gga-miR-489-3p	0.24	3.68×10^−6^	2.71×10^−4^	Down
gga-miR-499-5p	0.19	7.76×10^−5^	3.34×10^−3^	Down
gga-miR-1677-3p	0.16	1.36×10^−3^	3.01×10^−2^	Down
gga-miR-1563	0.15	7.89×10^−10^	1.75×10^−7^	Down
gga-miR-206	0.1	5.71×10^−14^	2.53×10^−11^	Down

*FC* fold change, *FDR* false discovery rate (corrected *P* value)

### Validation of miRNA expression using qRT-PCR

**Fig. 5:**
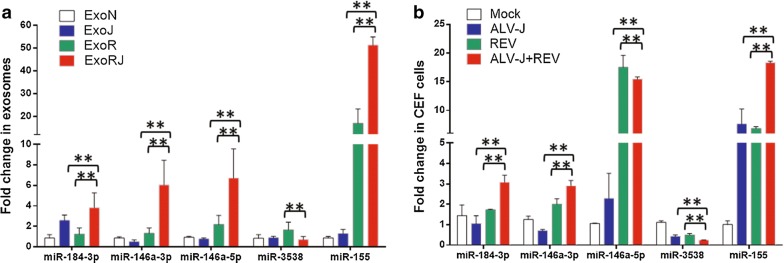
The qRT-PCR analysis for five miRNAs from exosomes and CEF cells. **a** The qRT-PCR results of the five miRNAs in exosomes were consistent with the sequencing. The data represent the mean ± SEM determined from three independent experiments (n = 3), with each experiment containing three technical replicates. Compared with the single-infection group: **P < 0.01. **b** The qRT-PCR results of four miRNAs in CEFs, including miR-184-3p, miR-146a-3p, miR-3538 and miR-155, were consistent with that in exosomes while miR-146a-5p expression in singly infected REV was significantly higher than co-infection with two viruses. The data represent the mean ± SEM determined from three independent experiments (n = 3), with each experiment containing three technical replicates. Compared with the single-infection group: **P < 0.01

To validate the results of deep sequencing, five miRNAs, including miR-184-3p, miR-146a-3p, miR-146a-5p, miR-3538 and miR-155 that changed significantly in the co-infection group compared to each single infection group, were selected for qRT-PCR analysis with primers in Table 4. After RNA was isolated from ExoN, ExoJ, ExoR and ExoRJ at 72 hpi, all 5 miRNAs showed expression profiles in CEF exosomes that were consistent with the small RNA sequencing data (Fig. 5a). Furthermore, the expressions of these five miRNAs were also verified in CEF cells co-infected with ALV-J and REV 72 hpi. Although the variation trends of miR-184-3p, miR-146a-3p, miR-3538 and miR-155 in both CEF cells and exosomes were consistent, some changes in each miRNA in CEF cells were less than in exosomes, indicating that the exosomes stably maintained these miRNAs (Fig. 5b).

**Table 4:** Primers used to detect miRNA expression using qRT-PCR

miRNA name	Mature sequences	Primer (5′–3′)
gga-miR-146a-5p	UGAGAACUGAAUUCCAUGGGUU	GTGAGAACTGAATTCCATGGGTT
gga-miR-146a-3p	ACCCAUGGGCUCAGUUCUUCAG	ACCCATGGGGCTCAGTTCTTC
gga-miR-184-3p	UGGACGGAGAACUGAUAAGGGU	TGGACGGAGAACTGATAAGGGT
gga-miR-3538	GUUCGGUGAUGAAACCAUGGA	GGTTCGGTGATGAAACCATGGA
gga-miR-155	UUAAUGCUAAUCGUGAUAGGG	GGTTAATGCTAATCGTGATAGGG
U6-F		CTCGCTTCGGCAGCACA
U6-R		AACGCTTCACGAATTTGCGT

### Target prediction

**Fig. 6:**
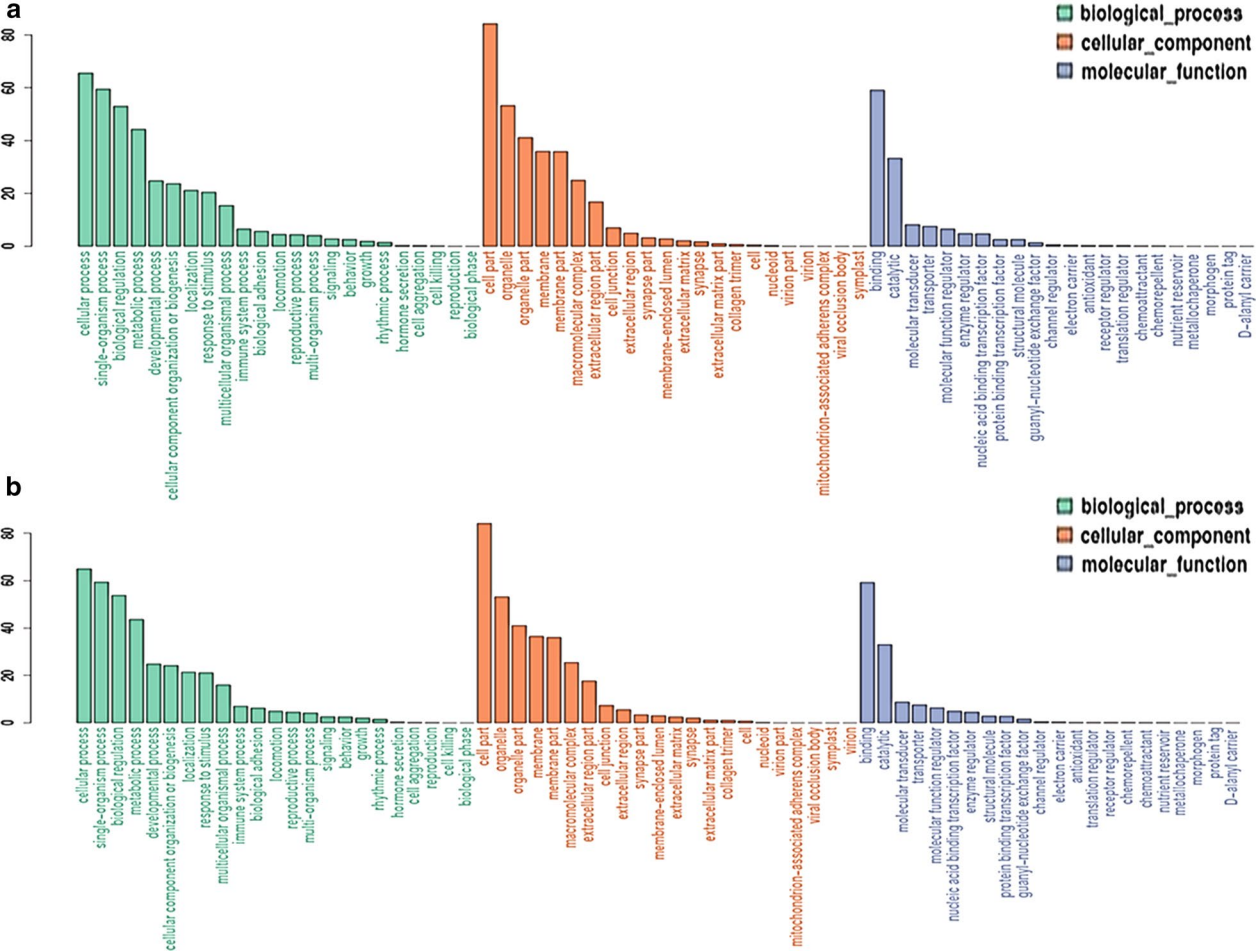
Gene Ontology (GO) annotation of predicted target genes from differentially expressed miRNAs by comparing co-infection with two viruses to single-infection ALV-J (**a**) and single-infection REV (**b**) groups

**Fig. 7:**
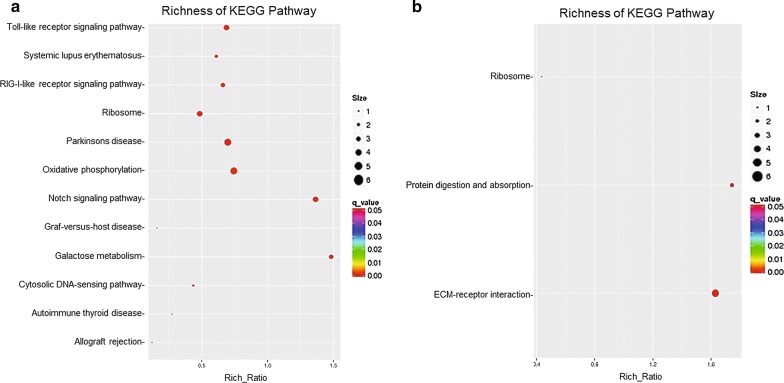
KEGG pathway analysis of predicted target genes from differentially expressed miRNAs by comparing co-infection with two viruses to single-infection ALV-J (**a**) and single-infection REV (**b**) groups

To understand the biological functions of the miRNAs identified in our analysis, miRanda was used to predict targets of the differentially expressed miRNA. Numerous target genes, 19,450 and 6058 for 54 and 16 miRNAs, respectively, were predicted as potential miRNA targets (Additional files 1 and 2). A GO annotation of the predicted target genes revealed that 100 and 35 target genes were significantly annotated for the 54 and 16 miRNAs, respectively, and they were involved in cellular processes, immune system processes, biology regulation, such as cytoskeleton organization, regulation of Ras protein signal transduction, ATP binding and guanyl-nucleotide exchange factor activity (Fig. 6, Additional files 3 and 4). To further analyse the roles that these miRNAs might play in regulatory networks, the putative miRNA targets were assigned to KEGG pathways using the KEGG GENES Database, PATHWAY Database and LIGAND Database. The results indicated that the most abundant KEGG terms were involved in the Toll-like receptor signalling pathway, oxidative phosphorylation, ribosome and other biological processes (Fig. 7, Additional files 5 and 6). In summary, these findings demonstrated that the differentially expressed miRNAs play important regulatory roles in virus-vector interaction, energy metabolism and cell growth.

## Discussion

In this study, an enhancement of viral transcription and protein expression was observed in ALV-J and REV co-infected CEF cells and reached a peak at 72 hpi. After Illumina small RNA deep sequencing of exosomes from CEFs co-infected with ALV-J and REV, a total of 54 (23 upregulated and 31 downregulated) and 16 (7 upregulated and 9 downregulated) miRNAs were identified by comparing the significantly differentially expressed miRNAs of ExoRJ with ExoJ and ExoR, respectively. Further, 5 miRNAs, including miR-184-3p, miR-146a-3p, miR-146a-5p, miR-3538 and miR-155, were verified by qRT-PCR and found to be consistent with the sequencing analysis. The analysis of the target prediction data demonstrated that these differentially expressed miRNAs participated in aspects of virus-vector interaction, oxidative phosphorylation, energy metabolism and cell growth in the process of co-infection with ALV-J and REV.

Useful as a biomarker, exosomes are overproduced by most proliferating cell types and contain a wide variety of microRNAs to induce a diverse range of functions, such as antigen presentation, cellular responses to environmental stresses, and propagation of pathogens [35–39]. Some miRNAs, such as let-7, miR-1, miR-15, miR-16, miR-151 and miR-375, which have roles in angiogenesis, haematopoiesis, exocytosis and tumourigenesis, have been reported in exosomes [40–44]. To further understand the relationship between exosomes and the parental CEF cells, miR-184-3p, miR-146a-3p, miR-146a-5p, miR-3538 and miR-155 expression was verified in both exosomes and CEF cells. In addition to miR-146a-5p, the qRT-PCR results in CEF cells of miR-184-3p, miR-146a-3p, miR-3538 and miR-155 were consistent with that in exosomes, suggesting that these miRNAs are key regulators in co-infection with ALV-J and REV.

In tumour progression, especially induced by viral synergistic infection, several studies have verified that miRNAs influence growth, differentiation, apoptosis, host-pathogen interactions, energy metabolism and other physiological processes [31–33]. While infecting cells, viruses integrate into the host genome to ensure viral persistence, which requires certain conditions for virus-vector interaction [45]. Simultaneously, the viral replication also benefits the cell’s transcriptional and translational machinery, which may enhance the growth of the host cells. In addition, these regulations are based on energy metabolism [46, 47]. Our data suggested that the significantly differentially expressed miRNAs between co-infected and two single virus infected CEFs participated in energy metabolism, virus-vector interaction and cell growth, suggesting that these miRNAs are key regulators in co-infection with ALV-J and REV. These initial findings will lead to further exploration of the mechanism of ALV-J synergistic infection with REV.

## Conclusion

We demonstrated that REV and ALV-J synergistically increased the accumulation of exosomal miRNAs. We revealed that a total of 54 and 16 miRNA genes were identified by comparing co-infection with two viruses with single-infected ALV-J and REV, respectively. These differentially expressed miRNAs participated in virus-vector interaction, oxidative phosphorylation, energy metabolism and cell growth, indicating potential new avenues to study the mechanism of synergistic infection of ALV-J and REV.

## Methods

### Cells and virus

DF-1 and chicken embryo fibroblasts (CEFs) cells were maintained in Dulbecco’s modified Eagle’s medium (DMEM) supplemented with 10% foetal bovine serum (FBS), 1% penicillin/streptomycin, and 1% l-glutamine, in a 5% CO_2_ incubator at 37°C. The stock SNV strain of REV at 10^3.2^ 50% tissue culture infectious doses (TCID_50_) and NX0101 strain of ALV-J at 10^3.8^ TCID_50_ were maintained in our laboratory. The TCID_50_ of the SNV and NX0101 strains were titrated by limiting dilution in DF-1 culture.

### Extraction of exosomes from CEF cells

The exosomes from Mock, single infection of ALV-J or REV, or co-infection of both ALV-J and REV CEFs were isolated using Total Exosome Isolation Reagent (Thermo Fisher Scientific) based on the manufacturer’s instructions.

### Transmission electron microscopy

The protocol was conducted as described in a previous study [48].

### Western blotting

CEF cells were lysed in cell lysis buffer (Beyotime) and incubated on ice for 5 minutes. ALV-J gp85, REV env expression, Hsp70, GRP78 and CD81 were detected by simple western analysis with anti-NX0101 gp85, anti-SNV env antibody, anti-Hsp70 (Bioss), anti-GRP78 (Bioss) antibody and anti-CD81 (Bioss) antibody at a 1:200, 1:200, 1:1000, 1:1000 and 1:1000 dilution, respectively.

### Illumina small RNA deep sequencing

Total RNA of the infected CEF exosome samples was separated by 15% agarose gels to extract the small RNA (18-30 nt). After precipitation by ethanol and centrifugal enrichment of the small RNA population, the library was prepared according to the method and process of the Small RNA Sample Preparation Kit (Illumina, RS-200-0048). The RNA concentration of the library was measured using Qubit® RNA Assay Kit in Qubit® 2.0 to preliminarily quantify and then dilute to 1 ng/µl. The insert size was assessed using the Agilent Bioanalyzer 2100 system (Agilent Technologies, CA, USA). The library with the expected insert size was then quantified accurately using TaqMan fluorescence probes of the AB Step One Plus Real-Time PCR system (library valid concentration >2 nM). The qualified libraries were sequenced by an Illumina HiSeq 2500 platform and 50 bp single-end reads were generated.

### Real-time quantitative reverse transcription polymerase chain reaction

Total RNA from CEF exosomes of either Mock, single infection of ALV-J or REV, or co-infection of both ALV-J and REV were isolated using the Tiangen RNeasy mini kit according to the manufacturer’s instructions, with optional on-column DNase digestion. RNA integrity and concentration were assessed by agarose gel electrophoresis and spectrophotometry. RNA (1 µg per triplicate reaction) was reverse transcribed to cDNA using the TaqMan Gold Reverse Transcription kit (Applied Biosystems). Real-time RT-PCR (qRT-PCR) was carried out using SYBR® Premix Ex TaqTM, and ALV-J or REV specific primers (Table 5). All values were normalized to the endogenous control GAPDH to control for variation. For qRT-PCR of miR-184-3p, miR-146a-3p, miR-146a-5p, miR-3538 and miR-155, we used a miRcute miRNA first-stand cDNA synthesis kit and a miRcute miRNA qPCR detection kit (SYBR Green) (TIANGEN). The reverse primer was provided in the miRcute miRNA qPCR detection kit as a primer complementary to the poly (T) adapter. Data were collected on an ABI PRISM 7500 and analysed via Sequence Detector v1.1 software. All values were normalized to the endogenous control U6 to control for variation. The specific primer for U6 was described in Table 4. Assays were performed in triplicate and average threshold cycle (CT) values were used to determine relative concentration differences based on the ΔΔCT method of relative quantization described in the manufacturer’s protocol.

**Table 5:** Primers used for real-time PCR

Gene	Primer	Primer sequence	Size of PCR product
REV	F	TTGTTGAAGGCAAGCATCAG	105 bp
	R	GAGGATAGCATCTGCCCTTT	
ALV-J	F	TGCGTGCGTGGTTATTATTTC	144 bp
	R	AATGGTGAGGTCGCTGACTGT	
GADPH	F	GAACATCATCCCAGCGTCCA	132 bp
	R	CGGCAGGTCAGGTCAACAAC	

### Target prediction

miRanda (http://www.microrna.org/microrna/) was used to predict targets of the differentially expressed miRNA. Gene enrichment and functional annotation analyses were conducted using Gene Ontology (GO; www.geneontology.org), Kyoto Encyclopedia of Genes and Genomes (KEGG, http://www.genome.jp/kegg), PATHWAY Database and LIGAND Database.

### Statistical analysis

Results are presented as the mean ± standard deviation(s). The T test and one-way ANOVA test was performed using SPSS 13.0 statistical software. A P value less than 0.05 was considered statistically significant.

## Additional files

**Additional file 1.** The potential miRNA targets of the differentially expressed miRNA between ExoRJ and ExoJ. Numerous target genes, 19,450 for 54 miRNAs, were predicted as potential miRNA targets.

**Additional file 2.** The potential miRNA targets of the differentially expressed miRNA between ExoRJ and ExoR. Numerous target genes, 6058 for 16 miRNAs, were predicted as potential miRNA targets.

**Additional file 3.** The GO annotation of the predicted target genes of the differentially expressed miRNA between ExoRJ and ExoJ. A GO annotation of the predicted target genes revealed that 100 target genes were annotated significantly for the 54 differentially expressed miRNAs between ExoRJ and ExoJ.

**Additional file 4.** The GO annotation of the predicted target genes of the differentially expressed miRNA between ExoRJ and ExoR. A GO annotation of the predicted target genes revealed that 35 target genes were annotated significantly for the 16 miRNAs between ExoRJ and ExoR.

**Additional file 5.** The KEGG analysis of the predicted target genes of the differentially expressed miRNA between ExoRJ and ExoJ. The KEGG analysis of the predicted target genes revealed that 7 regulatory networks were annotated significantly for the 54 differentially expressed miRNAs between ExoRJ and ExoJ.

**Additional file 6.** The KEGG analysis of the predicted target genes of the differentially expressed miRNA between ExoRJ and ExoR. The KEGG analysis of the predicted target genes revealed that 3 regulatory networks were annotated significantly for the 16 differentially expressed miRNAs between ExoRJ and ExoR.
